# Supplementary material for: Intrinsically disordered caldesmon binds calmodulin via the “buttons on a string” mechanism
Source: PeerJ. 2015 Sep 22;3:e1265. doi: 10.7717/peerj.1265 (PMC4582948; doi:10.7717/peerj.1265)
Supplement: Supplemental Information 1 [file peerj-03-1265-s001.docx]

SUPPLEMENTARY MATERIALS

Intrinsically disordered caldesmon binds calmodulin via the “buttons on a string” mechanism

Sergei E. Permyakov, ^†^ Eugene A. Permyakov, ^†^ and Vladimir N. Uversky^†,‡,*^

^†^Institute for Biological Instrumentation, Russian Academy of Sciences, 142290 Pushchino, Moscow Region, Russia;

^‡^Department of Molecular Medicine and USF Health Byrd Alzheimer's Research Institute, Morsani College of Medicine, University of South Florida, Tampa, Florida 33612, USA;

*To whom correspondence should be addressed: Vladimir N. Uversky, Department of Molecular Medicine, College of Medicine, University of South Florida, 12901 Bruce B. Downs Blvd, MDC3540, Tampa, FL 33612, USA; E-mail: [vuversky@health.usf.edu](mailto:vuversky@health.usf.edu)


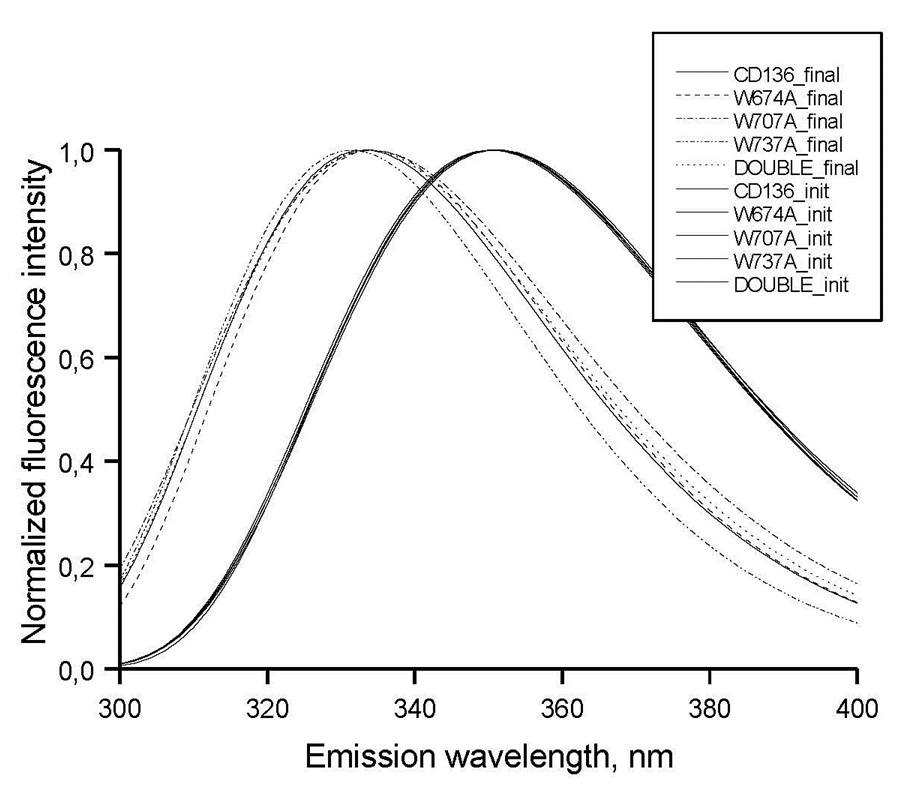


**Figure S1**. Normalized intrinsic tryptophan fluorescence spectra of wild type CaD_136_ and its mutants in the free and CaM-bound states. Proteins are characterized by almost indistinguishable fluorescence in their unbound forms, whereas binding to CaM differently affects intrinsic fluorescence spectra.

**Figure S2.** Difference spectra determined by the subtraction from the far-UV CD spectrum of the wild type CaD_136_ the far-UV CD spectrum of: W674A (**2**), W707A (**3**), W737A (**4**) and W674A/W707A (**5**). All measurements were carried out at a protein concentration of 0.6-0.8 mg/ml, cell pathlength 0.1 mm, 15^o^C.

**Figure S3.** Difference spectra determined by the subtraction from the near-UV CD spectrum of the CaD_136_ the near-UV CD spectrum of: W674A (**2**), W707A (**3**), W737A (**4**) and W674A/W707A (**5**). All measurements were carried out at a protein concentration of 0.6-0.8 mg/ml, cell pathlength 10 mm, 15^o^C.


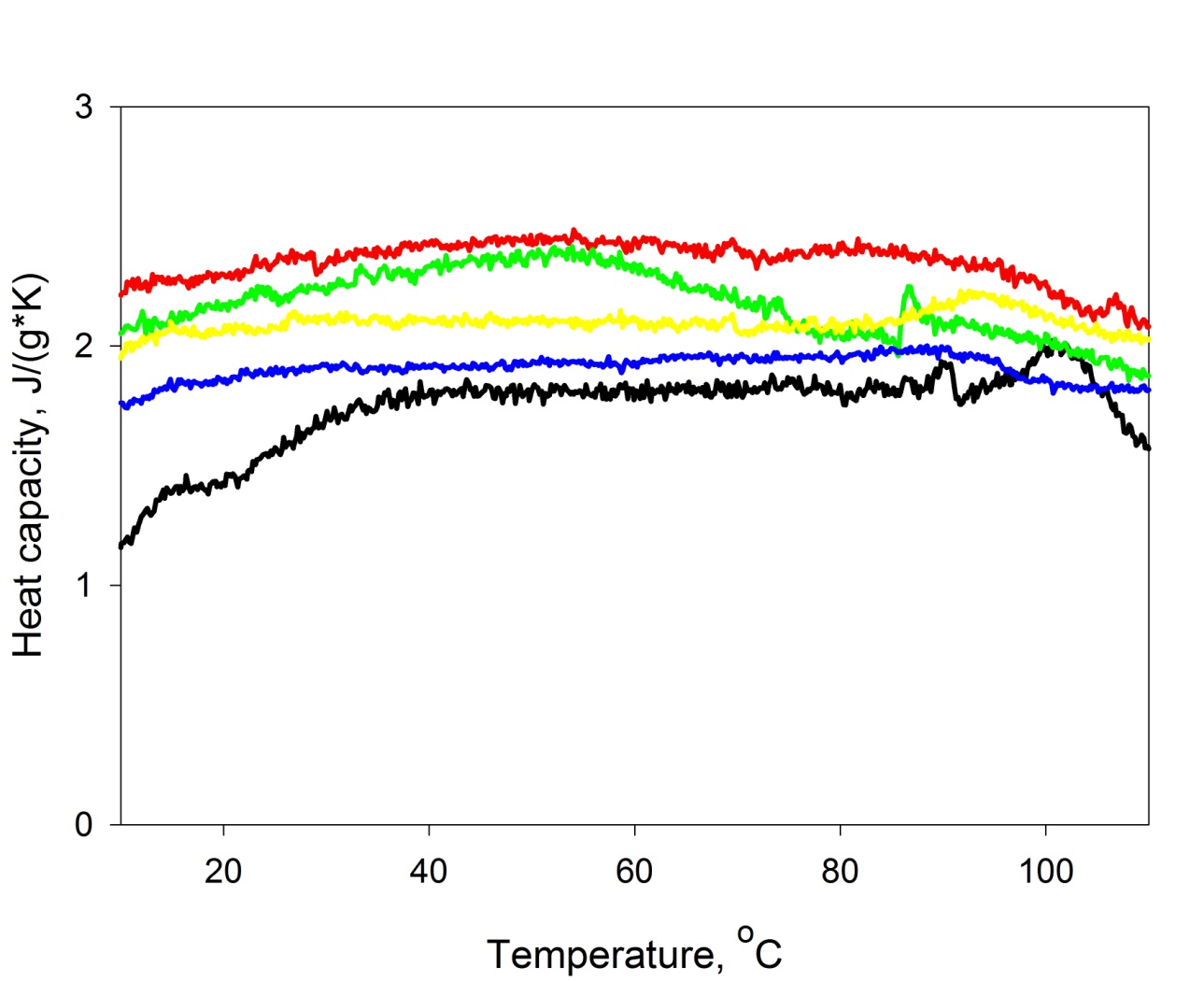


**Figure S4.** Calorimetric scans for wild type CaD_136_ and its mutants in solution. Experiments were performed in 50 mM H_3_BO_3_ buffer, pH 8.0. Protein concentrations were 0.97 mg/ml, 1.38 mg/ml, 1.21 mg/ml, 1.56 mg/ml and 1.96 mg/ml for the wild type (black curve), W674A (red curve), W707A (green curve), W737A (yellow curve), and W674A/W707A (blue curve), respectively.
